# Supplementary material for: Dual Targeting of Cancer Cells and MMPs with Self-Assembly Hybrid Nanoparticles for Combination Therapy in Combating Cancer
Source: Pharmaceutics. 2021 Nov 23;13(12):1990. doi: 10.3390/pharmaceutics13121990 (PMC8707712; doi:10.3390/pharmaceutics13121990)
Supplement: Supplementary file 1 [file pharmaceutics-13-01990-s001.zip › 1443866-supp-upload.pdf]

pharmaceutics

Supplementary Materials: Dual Targeting of Cancer Cells and MMPs with Self-assembly Hybrid Nanoparticles for Combination Therapy in Combating Cancer

Kai Zhang, Jingjing Li, Xiaofei Xin, Xiaoqing Du, Di Zhao, Chao Qin, Xiaopeng Han, Meirong Huo, Lei Yang and Lifang Yin

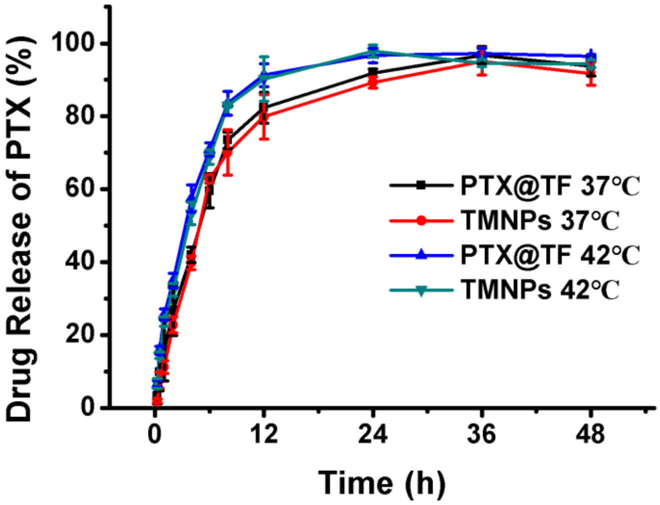

Figure S1. *In vitro* drug release of PTX in PTX@TF and TMNPs at 42°C or 37 °C. ($n = 3$).

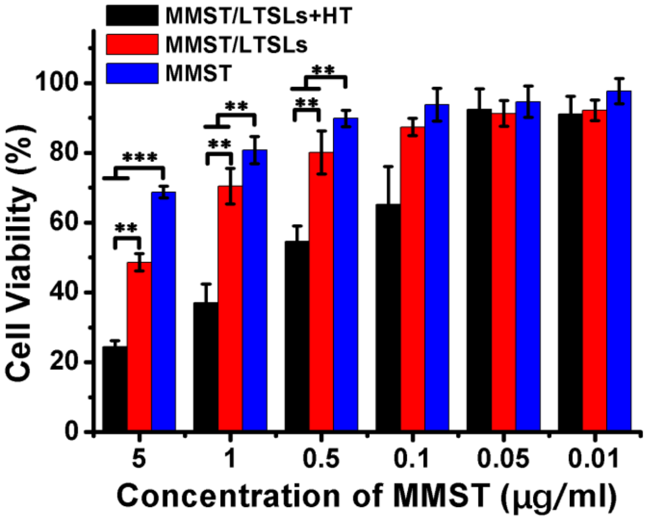

Figure S2. Cytotoxicity of MMST, MMST/LTSLs, and MMST/LTSLs+HT in 4T1 cells after a 48 h incubation at different concentrations (mean ± SEM, $n = 5$, $^{**}P < 0.01$, $^{***}P < 0.001$).

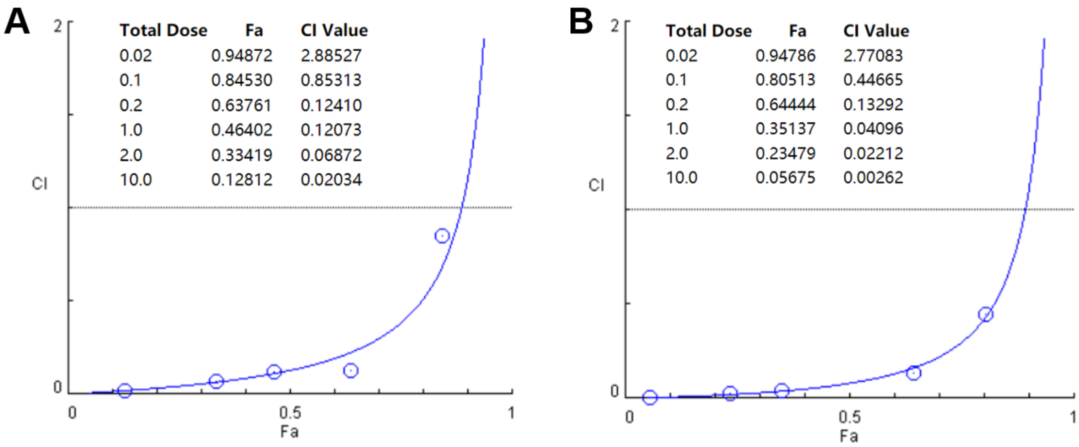

Figure S3. Combination index of (A) TMNPs and (B) TMNPs+HT at a mass ratio of PTX@TF : MMST/LTSLs=1 : 1. The cell inhibition rate (Fa) of the combined therapy index (CI) was calculated based on the cell survival rate.

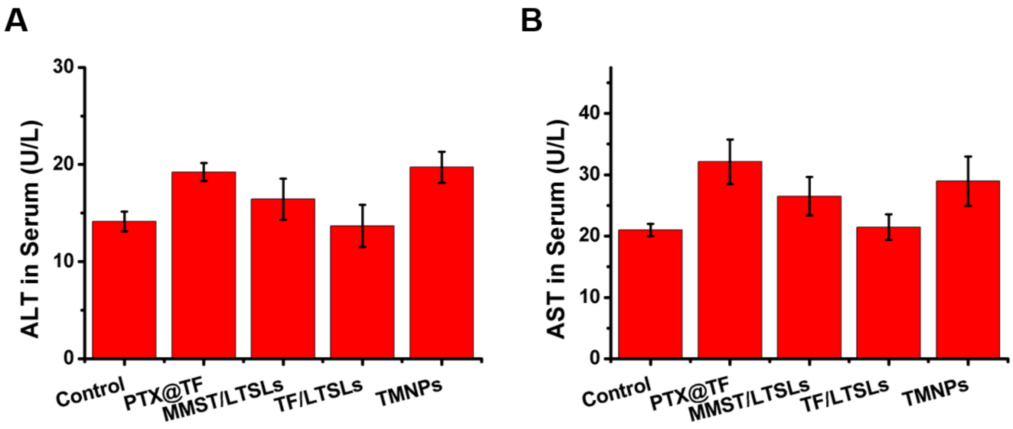

Figure S4. The levels of (A) ALT and (B) AST in serum. (mean ± SEM, $n = 3$).
